# Potentiation of *Scutellarin* on Human Tongue Carcinoma *Xenograft* by Low-Intensity Ultrasound

**DOI:** 10.1371/journal.pone.0059473

**Published:** 2013-03-25

**Authors:** Haixia Li, Haixia Fan, Zhu Wang, Jinhua Zheng, Wenwu Cao

**Affiliations:** 1 Laboratory of Sono- and Photo-theranostic Technologies, Harbin Institute of Technology, Harbin, Heilongjiang, China; 2 Department of Anatomy, College of Basic Medical Sciences, Harbin Medical University, Harbin, Heilongjiang, China; 3 Materials Research Institute, The Pennsylvania State University, University Park, Pennsylvania, United States of America; Dartmouth, United States of America

## Abstract

Scutellarin 7-*O*-β-d-glucuronide (*scutellarin*) has shown great potential as a chemotherapeutic agent for cancer treatment, but only at high dosage. Here we investigate the possibility of using low intensity ultrasound to reduce the *scutellarin* dosage. Ultrasound intensities of 1.0 W/cm^2^ and 0.05 W/cm^2^ were used for *in vivo* and *in vitro* experiments, respectively, and a very low dosage of *scutellarin* (15 nM) was used. Tumor-bearing Balb/c mice and SAS human-tongue squamous carcinoma cell suspensions were used for the *in vivo* and *in vitro* experiments, respectively. Each kind of subjects was divided into control, ultrasound-alone, *scutellarin-*alone, and combined ultrasound-*scutellarin* treatment groups. Only the combined treatment showed strong anticancer effects. In the *in vivo* case, the combined treatment significantly delayed tumor growth, initiated cellular chromatin changes (including a decrease in the number of cytoplasmic organelles and fragmentation of condensed nuclear chromatin), inhibited tumor angiogenesis and lymphangiogenesis, stopped cancer-cell proliferation, decreased MMP-2 and MMP-9 expression levels and caused cancer-cell apoptosis. In the *in vitro* case, the combined treatment produced cancer cell-shape irregularity in a manner seriously fractured microvilli, inhibited cancer-cell migratory and invasion activities, and induced cancer-cell apoptosis. Because the combined treatment did not increase intracellular ROS production, *scutellarin* is not a sonosensitizer so that the anticancer effect is not through sonodynamic therapy. Low-intensity ultrasound is merely increasing the permeability of *scutellarin* into cancer cells. Based on our results, one may perform localized chemotherapy using much reduced dosage of the drug with the help of low intensity ultrasound, which will greatly minimize side effects.

## Introduction

Oral squamous cell carcinoma (OSCC) is a common cancer of the head and neck region, characterized by an aggressive growth pattern, high degree of local invasiveness and cervical lymph node spread [1]–[2]. Despite recent advances in diagnostic methods and treatment strategies, the overall survival rate of patients with OSCC has not significantly improved. The poor prognosis of patients with OSCC, whose 5-year mortality rate is over 50% [1], is due to the disease’s tendency of strong local invasion and distant metastasis [3].

In terms of treatment, surgery is still the most common form of initial definitive treatment for the majority of OSCC patients [4], chemotherapy is a treatment option for patients with advanced OSCC, while radiotherapy is commonly used for the management of early-stage and locally advanced OSCC [5]. Induction chemotherapy generally provides local treatment or palliative therapy for patients with recurrent and/or metastatic disease [6]. These forms of treatment usually yield many side effects. In particular, surgical treatment may require organ removal in the oral cavity, resulting in oral dysfunction, such as difficulty in eating, swallowing, and speaking.

Due to their non-selective killing of malignant and normal cells, both radiotherapy and chemotherapy may seriously degrade the quality of life for patients. Although combination radiotherapy–chemotherapy treatment is believed to yield additional benefits, it still does not reduce side effects. Moreover, radical radiotherapy and/or chemotherapy have limited application for patients with recurrent disease or may produce another primary manifestation in the irradiated field. Hence, alternative technologies or complementary treatment methods are constantly in demand, among which the most desirable ones are non-invasive therapies that could provide localized treatment to tumors with much less side effects [7].

Low-level ultrasound sonication has been found to induce reversible changes in membrane permeability and improve drug-delivery efficiency [8]–[12]. As such, low-level ultrasound therapy has been used to potentiate the cytotoxicity of chemotherapeutics [13]–[14] and improve drug delivery efficiency by sonopermeabilization, particularly by increasing the permeability of the brain blood-vessel barrier [15]. Moreover, because the effects of sonication are localized to pathological sites, it can minimize the damage to surrounding normal tissues. In recent years, ultrasound has become a preferred clinical technique in the regulation of targeted therapy [16]–[18].

Due to the highly disorganized nature of tumor vasculature, tumor tissue is characterized by high blood pressure and high blood viscosity, which cause difficulties in drug administration to tumor sites. Sonication not only increases membrane permeability and intracellular drug uptake via the generation of small holes on the cell membrane by cavitation-induced jet-stream but also produces noncavitating mechanical effects that can increase drug concentration at tumor sites and enhance intracellular uptake [8], [19]. By such means, sonication can enhance the overall antitumor effects of chemotherapy by promoting drug delivery to tumor sites and increase the permeability of cancer-cell membranes.

Studies have shown that *scutellarin* can play a beneficial biological role in numerous mammalian systems, including free-radical scavenger, cell-apoptosis inhibitor, as well as anti-inflammatory, antitumor, and antimutagen agent [20], [21]. In our previous studies, we demonstrated that s*cutellarin* significantly inhibits the growth, adhesion, and migration of SAS/HSC-4 tongue-cancer cells [22] without significant toxicity on normal cells at lower concetration[23], indicating its excellent potential as a chemotherapeutic agent. It was also shown that the effectiveness depends on the concentration of s*cutellarin.* No significant effects were found if the concentration is less than 50 nM. In this study, we examined the effect of treatment consisting of a much lower concentration of *scutellarin* (15 nM) combined with low-level ultrasound as a stimulus. The purpose was to verify whether ultrasound can enhance the antitumor effects of a low concentration of *scutellarin* and to identify the possible mechanism responsible for the synergistic antitumor effect of ultrasound-*scutellarin* therapy.

## Materials and Methods

### Tumor Model and Chemicals

Four- to five-week old male Balb/ca nude mice (SLAC; Shanghai Laboratory Animal Center, Shanghai, China) were housed under specific-pathogen free (SPF) conditions. To create an animal *xenograft* model of human-tongue squamous carcinoma, a 0.2-mL suspension of SAS human-tongue squamous carcinoma cells (8×10^4^ cells/mL; Human Science Research Resources Bank, Osaka, Japan) in a serum-free medium was subcutaneously injected into the back flank of each mouse. *Scutellarin* of 99% purity was purchased from Beidouxing Pharmaceutical Co. Ltd. (Tianjin, China). All *in vivo* experimental procedures were approved by the Laboratory Animal Committee of the Harbin Medical University (Harbin, China).

**Figure 1:**
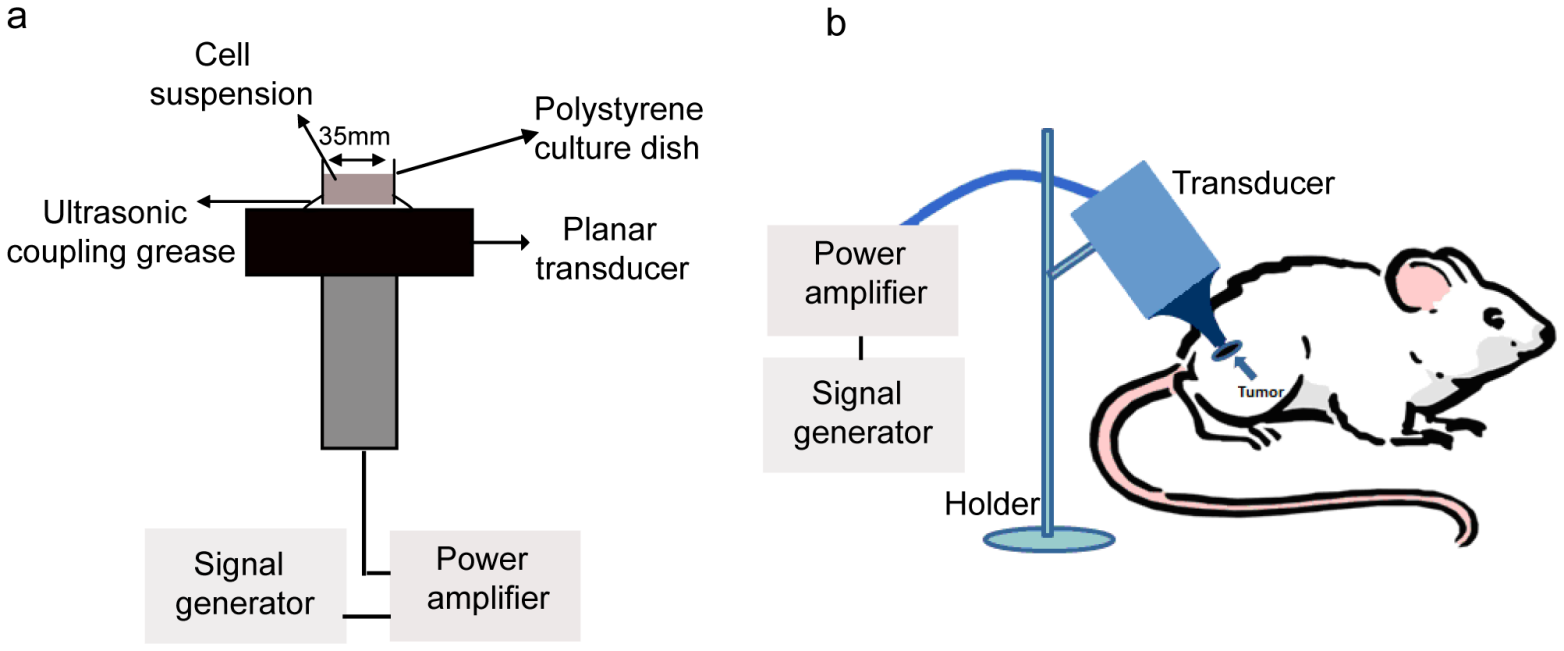
Schematic diagram of sonication devices for the *in vitro* and *in vivo* experiments. (a) The tone-burst ultrasonic transducer (1.0 MHz center frequency, 10% duty factor) was fixed by aluminum stents facing upward. The culture dish was placed above the center of the transducer for the *in vitro* experiments. (b) The tone-burst ultrasound signal was applied through a tapered aluminum buffer head with its front surface directly in contact with the skin above the tumor site for the *in vivo* experiments.

Fig. 1 shows the ultrasound treatment system developed by the Condensed Matter Science and Technology Institute, Harbin Institute of Technology (Harbin, China). In the *in vitro* experiments, a transducer was fixed with aluminum metal stents such that the tone-burst ultrasonic transducer (4.0 cm diameter, 1.0 MHz center frequency, 10% duty factor, 100 Hz repetition frequency) faced upward. The culture dish (3.5 cm diameter) was placed in the center of the transducer (Fig. 1a). In the *in vivo* experiments, a tone-burst ultrasound signal generated by a 3.0 cm-diameter piezoelectric transducer with center frequency of 1.0 MHz was applied through a tapered aluminum buffer head whose front surface (5 mm diameter) was directly in contact with the skin above tumor site through ultrasonic coupling grease (Fig. 1b). Ultrasound intensity was measured in degassed water using a calibrated hydrophone (HN-1000, Ondam, USA) [24]. A thermocouple was used to ensure that the temperature in the cell suspensions and the surface of the aluminum buffer head increased less than 2°C in all experiments.

The mice were randomly divided into 4 groups: 1) untreated control group (C group, n = 10); 2) low-intensity ultrasound treatment group [Us group, 1.0 W/cm^2^ intensity, 1 MHz frequency, 10% duty factor, 15 min duration; n = 12]; 3) the oral gavage of *scutellarin* group [S group, dosage of 10 mg/kg on days 1, 3, 5, 8, 10, 12, 15, and 17; n = 12]; 4) low-intensity ultrasound and oral gavage of *scutellarin* group [Us+S group, *scutellarin* administration 1 h before ultrasound treatment; n = 12] and the *in vivo* treatment began when the tumor size of the mouse models reached about 0.3 to 0.4 cm in diameter after 10 days of implantation. SAS human-tongue squamous carcinoma cells were sonicated at intensities of 0.01, 0.03, 0.05, 0.08, 0.1, and 0.12 W/cm^2^ for 1, 2, or 3 min (1 MHz tone-burst frequency, 10% duty cycle), respectively, before being collected and analyzed using trypan-blue staining to detect immediate injuries by ultrasound.

The tumor diameters in the short (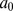) and long (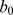) axes were measured for calculation of initial tumor volume (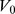) according to the following formula [25]:
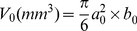
(1)


The growth of tumors was quantified using the relative volume percentage 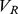:
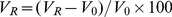
(2)


At the end of the treatment period, all tumors were excised for analysis.

In the *in vitro* experiments, SAS human-tongue squamous carcinoma cell or rat myoblast line (L6) (Wuhan Boster Bio-engineering Limited Company, Wuhan, China) suspensions were also divided into 4 groups as mentioned above. The samples were prepared by seeding 1 million cells in 1 ml of medium in a 3.5-cm dish, followed by incubation at 37°C for 12 h. Another 1 ml of fresh medium, with or without *scutellarin*, was added at the time of the experiment. After adding *scutellarin* to the S and Us+S groups, the dishes were incubated at 37°C for 30 min. Then sonication was conducted at 0.05 W/cm^2^ for 1 min to the Us and Us+S groups, and the dishes were incubated at 37°C again before subjected to analyses.

### Cell Viability and Growth

Cell viability after each treatment was evaluated by trypan-blue exclusion test. The test was performed by mixing 200 µL of cell suspension with an equal amount of 0.4% trypan blue solution (Sigma Aldrich, St Louis, MO, USA) in phosphate-buffered saline (PBS) and incubating the mixture at room temperature for 3 min. The number of viable cells was determined by counting the number of cells excluding trypan blue using an ECLIPSE TS100 optical microscope (Nikon, Tokyo, Japan) with a hemocytometer. Cell growth was evaluated by MTT assay at 8, 24, 48, 72, and 96 h after each treatment using a method similar to our previous report [22]. Each group contained 6 replicate wells, and each experiment was repeated 3 times.

### Wound-healing Assay

SAS cells (1×10^4^) were seeded into 24-well cell culture plates and cultured to 80% confluence. After treatments, cells were washed 3 times with PBS. A cell wound was created with a 200-µL micropipette tip and imaged at ×100 magnification (Nikon Eclipse TS100, Japan). Treated cells with cell wounds were cultured in 0.4% (v/v) heat-inactivated fetal bovine serum (FBS) medium for 8 h, 24 h, or 48 h and then imaged using optical microscopy.

### Cell-migration and Invasion Assay

Cell migration and invasion were assessed using Millicell® Hanging Cell Culture Inserts (8-µm pore size; Millipore, USA) after each treatment. Cell-migration assay was performed by incubating the inserts at 37°C with 5% CO_2_ for 24 h. Cell invasion assay was performed by coating the upper surfaces of each insert with Matrigel before incubation at 37°C with 5% CO_2_ for 48 h. Cell migration and invasion were quantified by counting the number of cells in 10 visual fields on the lower surface of each filter using phase-contrast microscopy.

### Scanning Electron Microscopy

Cells were prepared for scanning electron microscopy (SEM) by fixation with 2.5% glutaraldehyde in 0.1 M of PBS (pH 7.2 to 7.4) at 24 h after sonication (0.05 W/cm^2^ intensity, 1 MHz tone-burst frequency, 1-min duration). The treated cells were post-fixed in 1% osmium tetroxide (OsO_4_), washed with PBS, dehydrated with graded alcohol, displaced, and dried at the critical point. A thin layer of gold was evaporated onto the surface before observation using a scanning electron microscope (S-3400N, Hitachi, Japan).

### Measurement of Intracellular ROS

The generation of intracellular reactive oxygen species (ROS) was examined using a ROS assay kit (Applygen, Beijing, China). Briefly, after each treatment, cells were incubated with 10 µM of DCFH-DA for 20 min at 37°C in the dark. ROS generation was then determined by fluorescence microscopy (Olympus BX51, Japan) or flow cytometry (BD Biosciences) at an excitation wavelength of 488 nm and an emission wavelength of 525 nm. More than 3 sampling fields were observed by fluorescence microscopy and 10,000 cells were analyzed with flow cytometry in each measurement. The extent of ROS generation was calculated based on the mean fluorescence, as determined by flow cytometry, and the data presented in a histogram.

### Transmission Electron Microscopy

For transmission electron microscopy (TEM) study, xenografts were dissected and fixed with 2.5% glutaraldehyde for 2 h, post-fixed in 1% OsO_4_ at 4°C for 2 h, and embedded with Epon812 (EM Sciences, Washington, PA, USA) for 72 h at 60°C. Ultrathin sections were cut and stained with uranium acetate, followed by lead citrate, and then observed under a transmission electron microscope (JEOL 200, Hitachi, Japan).

### TUNEL Assay for Apoptotic Cells *in vivo*

To identify apoptotic cells *in vivo*, the staining of 4-µm thick paraffin-embedded sections was evaluated using a terminal deoxyribonucleotide transferase-mediated nick-end labeling (TUNEL) assay kit (Roche, Switzerland) according to the manufacturer’s instructions. The extent of apoptosis was evaluated by counting the number of TUNEL-positive (brown-stained) cells. The apoptotic index was calculated as the number of TUNEL-positive cells divided by the total number of cells in 10 randomly selected high-power fields (magnification × 200).

### Immunohistochemical Staining

Deparaffinized sections taken from each tumor were incubated with 1% BSA for 30 min and then stained with mouse monoclonal anti-MMP-2 (sc-13595; 1∶200; Santa Cruz Biotechnology, Inc., Santa Cruz, CA, USA), goat polyclonal anti-MMP-9 (sc-6840; 1∶200; Santa Cruz Biotechnology), mouse monoclonal anti-PCNA (sc-25280; 1∶200; Santa Cruz Biotechnology), rat monoclonal anti-CD105 (sc-101443; 1∶200; Santa Cruz Biotechnology), and rabbit polyclonal anti-Lymphatic Endothelial Marker D2–40 (bs-7180R; 1∶200; Beijing Biosynthesis Biotechnology Co., LTD, Beijing, China). Cytoplasmic staining was scored positively for MMP-2, MMP-9, and CD105, and nucleus staining was scored positively for PCNA and D2–40 (× 400 magnification). The extent of cell proliferation was evaluated by counting the number of PCNA-positive cells in 10 randomly selected high-power fields and the proliferation index was calculated as the number of PCNA-positive cells divided by the total number of cells in the fields. For MMP-2 and MMP-9 analysis, 10 areas were randomly selected under a microscope at a magnification of 200. Image Pro Plus 6.0 (Media Cybernetics, Inc., Bethesda, MD, USA) was used to quantify the extent of immunopositive expression in cells with integrated optical density (IOD) values. Determination of microvessel density (MVD) and lymphatic vessel density (LVD) was independently performed by 2 investigators according to methods described by Weidner et al. [26]. Sections were scanned under light microscopy at low magnification (×100), tissue areas with the greatest number of distinctly highlighted microvessels and lymphatic vessels (hot spots) were selected, and MVD and LVD were determined by counting all stained vessels at high magnification (×200) in 5 fields for each tumor.

### Statistical Analysis

All experiments were independently performed at least 3 times. Data were analyzed using one-way ANOVA and are presented as mean ± standard deviation (SD) values. Values that reached a p<0.05 level of significance were considered statistically significant.

## Results

### Cell Viability and Growth

**Figure 2:**
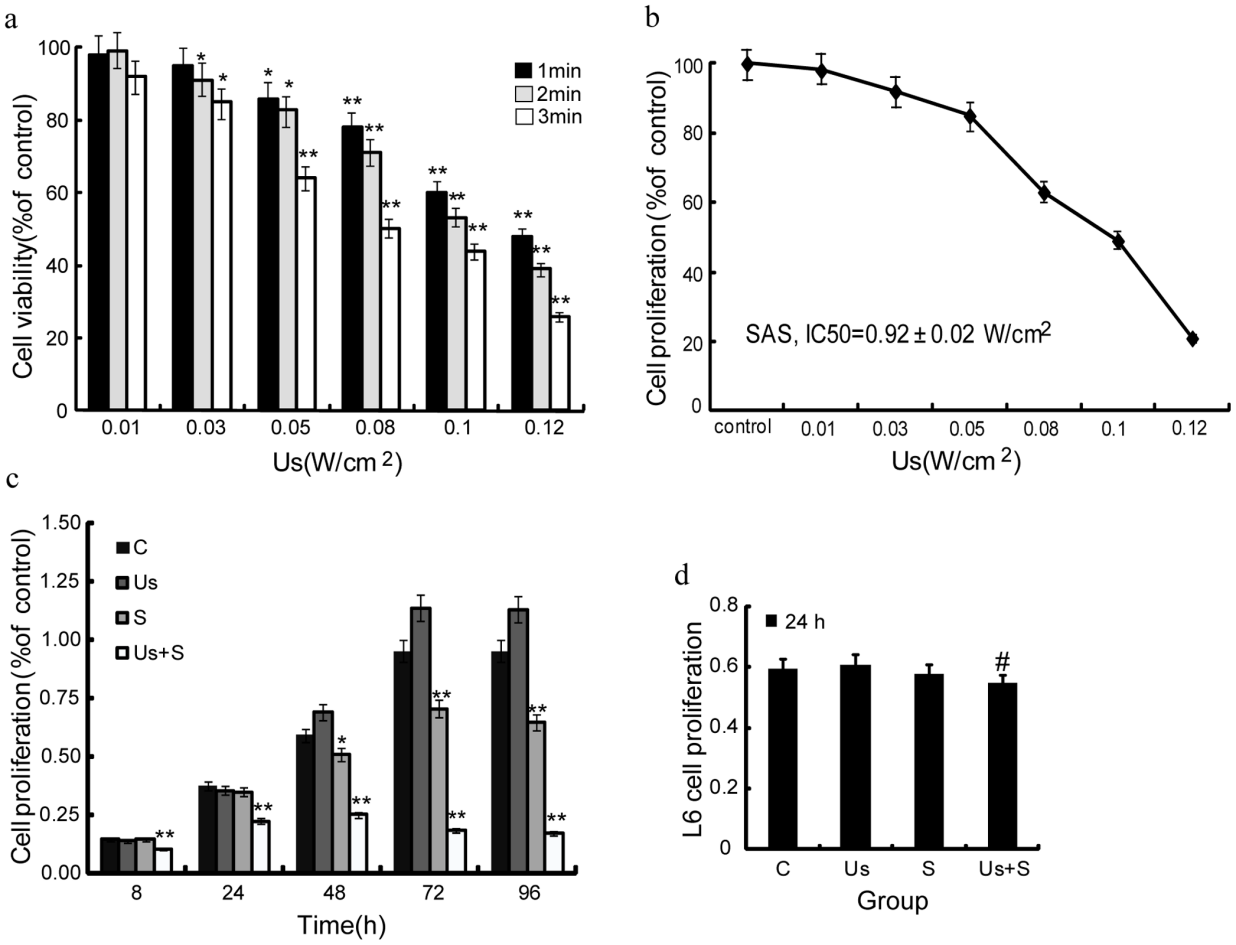
Effect of ultrasound and *scutellarin* on SAS or L6 cell viability. (a) Cell viability evaluated by trypan-blue exclusion test 1 min after sonication at intensities of 0.01, 0.03, 0.05, 0.08, 0.1, and 0.12 W/cm^2^ for 1, 2, or 3 min. (b) Cell growth evaluated by MTT assay 24 h after sonication at various intensities for 1 min, shown by absorbance at 490 nm. (c) Cell growth evaluated by MTT assay 8, 24, 48, 72, and 96 h for C, Us, S, and Us+S groups. For the Us+S treatment, cells were exposed to 15 nM of *scutellarin* for 30 min and then sonicated at 0.05 W/cm^2^ for 1 min. (d) L6 cell growth evaluated by MTT assay 24 h for C, Us, S, and Us+S groups. For the Us+S treatment, cells were exposed to 15 nM of *scutellarin* for 30 min and then sonicated at 0.05 W/cm^2^ for 1 min. Values are mean ± SD (n = 6). Statistical significance was determined by one-way ANOVA Dunnett’s t-test. C: control group, Us: low-level-ultrasound treatment group, S: *scutellarin* treatment group, Us+S: low-level-ultrasound-combined-with-*scutellarin* treatment group. *p<0.05; **p<0.01 *vs.* control group; # no significance *vs.* control group.

The results of trypan-blue staining revealed that sonication caused significant SAS cell injury in an intensity- and time-dependent manner (Fig. 2a). MTT assay indicated that sonication also inhibited SAS cell growth in an intensity-dependent manner (Fig. 2b). The IC50 value was calculated to be 0.92±0.02 W/cm^2^, and the cell survival rate to be 85% at 24 h after sonication (0.05 W/cm^2^ intensity, 1 min duration, 1 MHz tone-burst frequency, 10% duty cycle). The effects of *scutellarin*-alone or combined *scutellarin*-and-ultrasound (0.05 W/cm^2^ intensity, 1 min duration, 1 MHz tone-burst frequency, 10% duty cycle) on SAS cells were studied. S*cutellarin-*alone treatment was found to moderately inhibit cancer cell growth after 48 h, but the combination treatment had significantly inhibited cell growth after 8 h in a time-dependent manner (Fig. 2c). In contrast, low-level ultrasound-alone treatment not only failed to inhibit cell growth, but actually promoted cell proliferation (Fig. 2c). These results indicate that low-intensity ultrasound can significantly enhance *scutellarin*-induced cell damage and cell-growth inhibition, whereas low-level ultrasound alone actually increases total cell proliferation after 48 h.

In addition, MTT assay was used to detect the therapeutic index of this approach for a type of normal cells (L6 rat myoblasts). The results show that the ultrasound alone, *scutellarin* alone or combined treatment failed to show significant cytotoxicity on L6 rat myoblasts (Fig. 2d).

### Scanning Electron Microscopy

**Figure 3:**
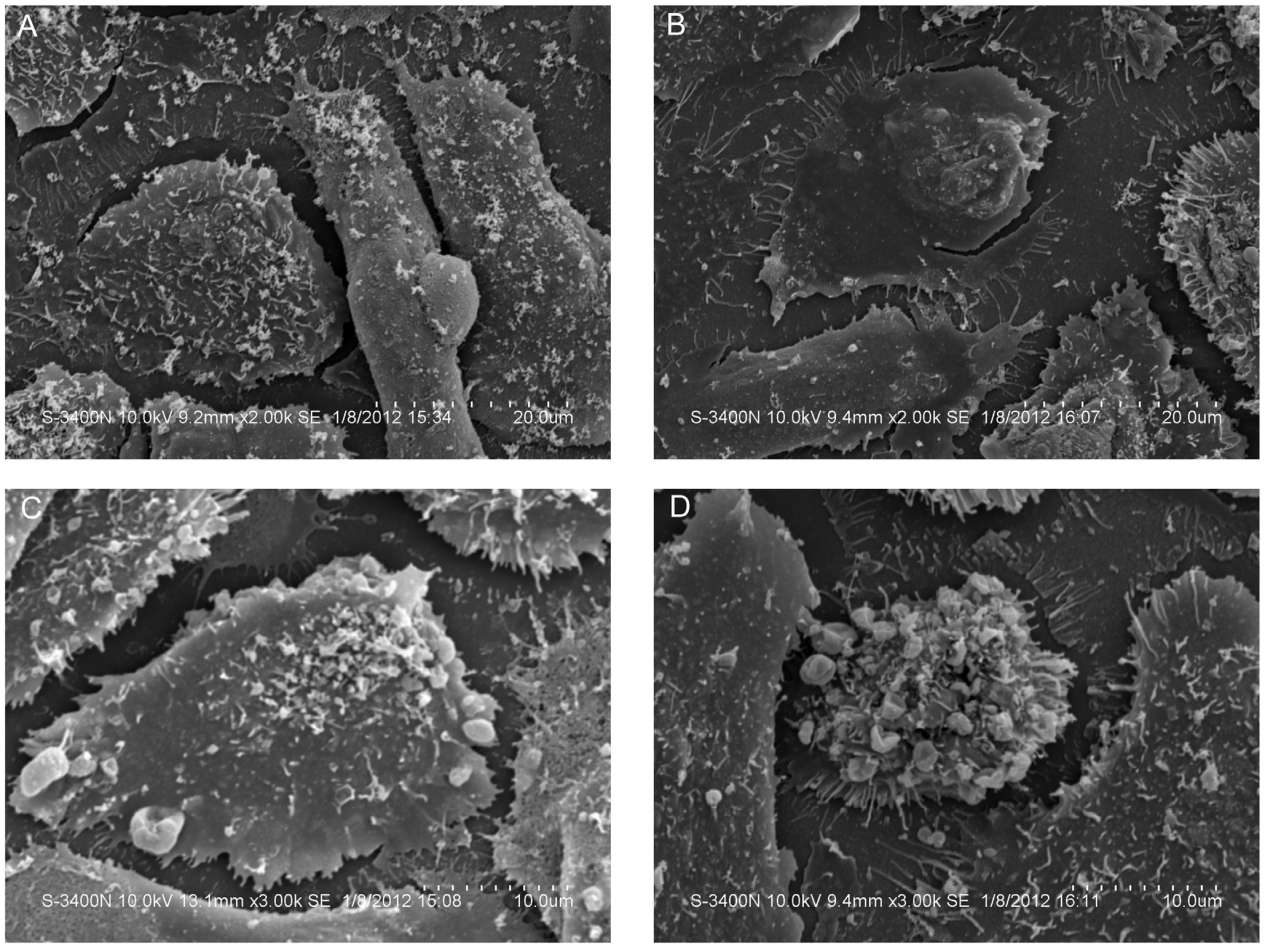
Scanning electron microscopy of SAS cells. (a) Control cells. (b) Cells after sonication at 0.05 W/cm^2^ for 1 min. (c) Cells treated with 15 nM of scutellarin. (d) Cells treated with 15 nM of *scutellarin* followed by sonication at 0.05 W/cm^2^ for 1 min. Scale bar = 20 µm.

Examination of morphological changes and apoptosis by SEM 1 h after sonication revealed that nuclear structure of the cells in the C group was clear, with the cells having either a round or oval shape and numerous microvilli and ruffles (Fig. 3a). In contrast, several cells in the Us group had relatively fewer microvilli and showed characteristics of early apoptosis (Fig. 3b). Likewise, several cells in the S group had significantly fewer microvilli, and several blebs or granular protrusions had emerged on the cell surface (Fig. 3c). On the other hand, the cells in the Us+S group had not only become irregular in shape with seriously fractured microvilli but had also become attached to several spherical bodies with a smooth surface, which were identified as typical apoptotic bodies (Fig. 3d).

### Cell Invasion and Migration

**Figure 4:**
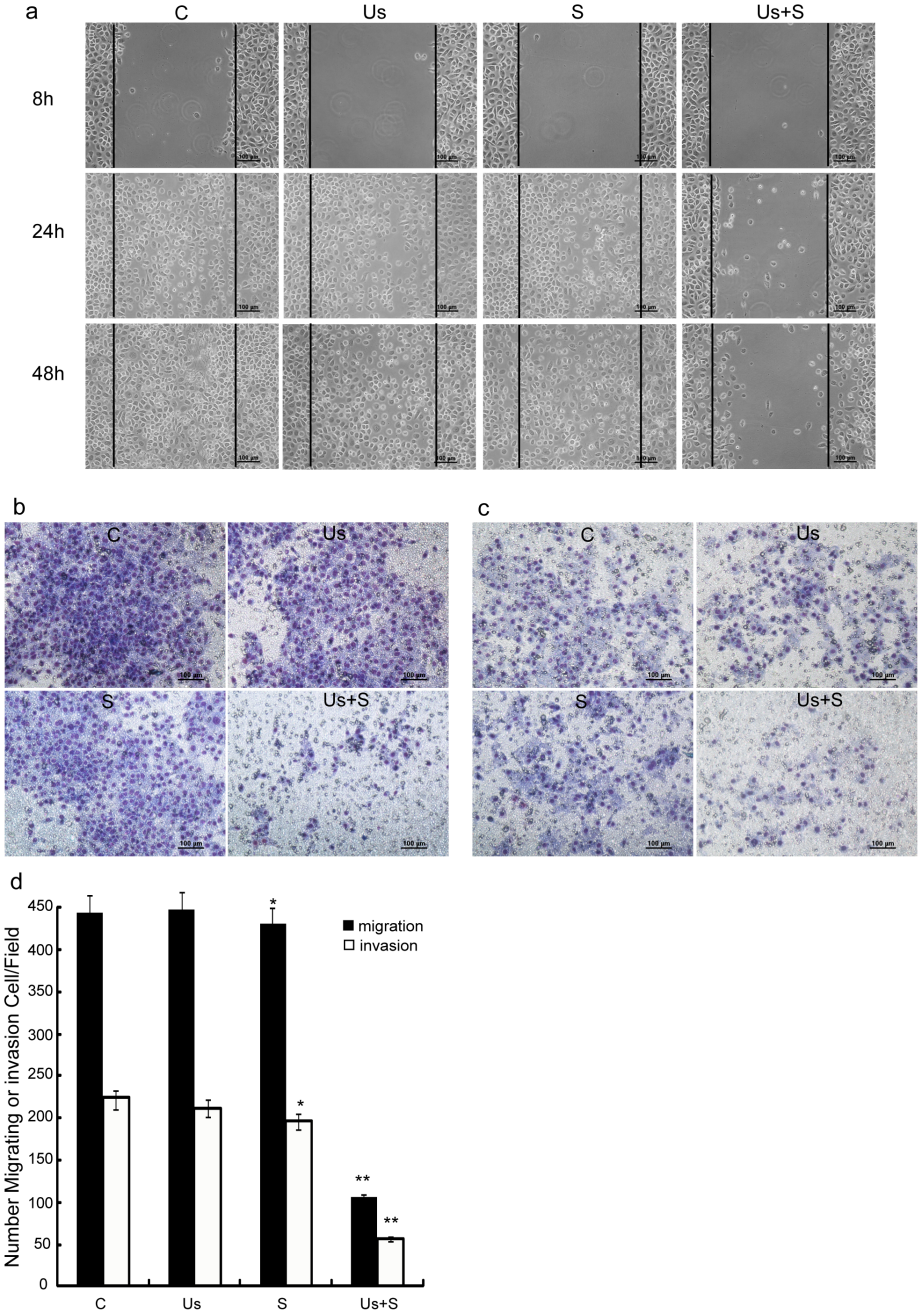
Inhibition of SAS cell migration and invasion by *scutellarin* and ultrasound. (a) Wound healing assay (scale bar = 100 µm) for C, Us, S, and Us+S groups. (b) and (c): Effect of S and Us treatments on SAS cell migration after 24 h (b) and invasion after 48 h (c), respectively, as assessed by transwell migration and invasion assay. (d) Number of stained cells after treatment. The average of 10 fields was counted in 3 replicate studies for each cell line. Data are mean ± SD values of 3 experiments (n = 3). Statistical significance was determined by one-way ANOVA Dunnett’s t-test. *p<0.05; **p<0.01 *vs.* control cells.

Our experiments indicated that the combined treatment enhances the cell-damage capacity of *scutellarin* by increasing inhibition of cancer cell growth and inducing cell apoptosis. To assess cell migration and invasion activities, wound-healing assay and Transwell assay were subsequently performed. As shown in Fig. 4a, the wound edges of cells in the C group were barely visible after culturing for 24 h, and became completely defused after culturing for 48 h. The migration behaviors of cells in the Us and S groups were not significantly different from those of the C group after 48 h. However, cells in the Us+S group experienced no migratory activity, nor did the wound edges experience any sign of diffusion, leading to an increase in wound size. Validation of these results (Fig. 4b, d) confirmed that cells in the Us+S group experienced significantly less migratory activity than the other groups (p<0.01). In addition, cells in the Us+S group showed very little cell invasion (Fig. 4c, d; p<0.01), while no significant differences were found in terms of cell invasion among the other 3 groups.

### Intracellular Reactive Oxygen Species Production

**Figure 5:**
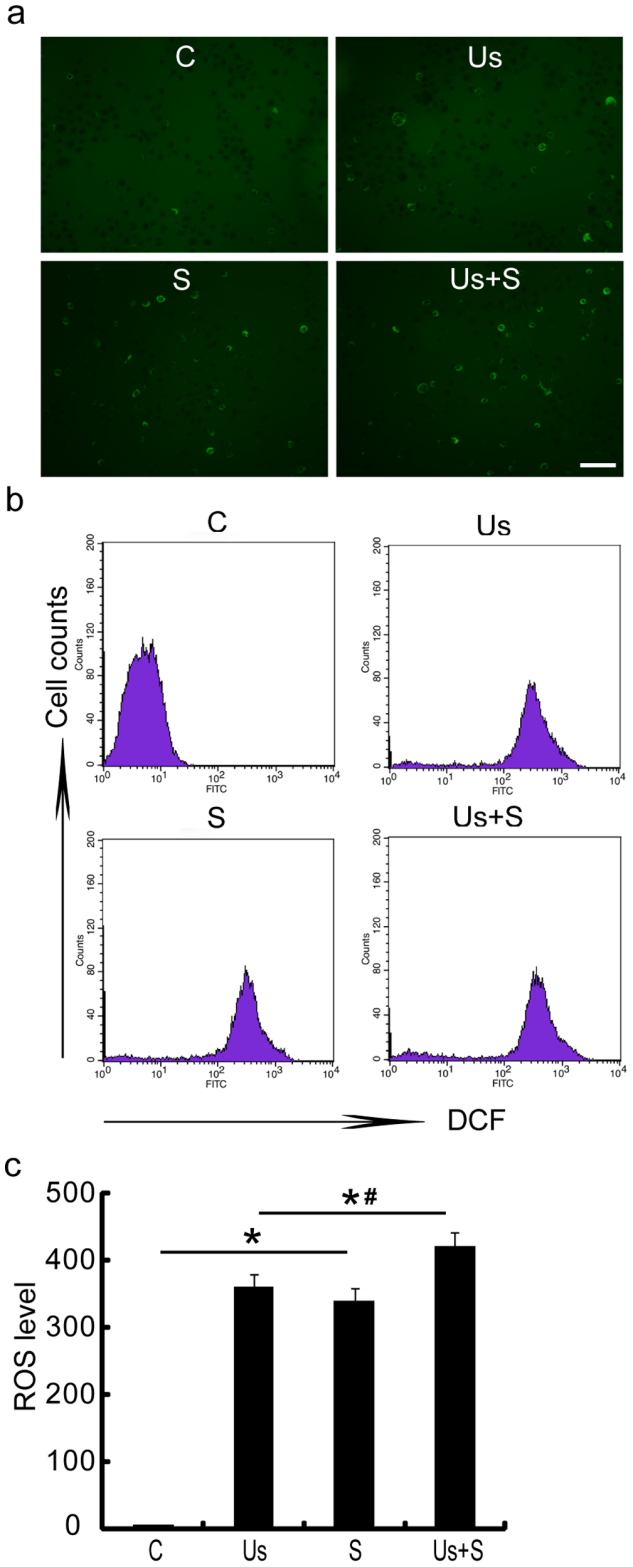
Induction of reactive oxygen species (ROS) generation in SAS cells by *scutellarin* and ultrasound. (a) Intracellular ROS production was observed under fluorescence microscopy by 2′,7′-dichlorofluorescin diacetate (DCFH-DA) staining for the C, Us, S, and Us+S groups. Light green fluorescence shows ROS levels in the cytoplasm. Scale bar = 100 µm. (b) Cells were stained with DCFH-DA and analyzed by flow cytometry. (c) Relative ROS levels in terms of mean fluorescence are shown as ratios compared to the C group. Data shown are the mean ± SD values calculated from representative measurements obtained from at least 3 independent experiments. Statistical significance was determined by one-way ANOVA S-N-K test. *p<0.01 vs. control; *# p<0.05 vs. Us+S groups. The increase in ROS generation for Us+S is much less than sonodynamic effect.

To examine the mechanism by which ultrasound enhances the cytotoxicity of chemotherapy drugs, the intracellular ROS level was measured to determine whether sonication enhances *scutellarin*-induced production of ROS. The results revealed that both the S and the Us groups experienced increased ROS generation compared with the C group (Fig. 5, p<0.01); and there is a moderate increase of ROS in the case of combined ultrasound+scutellarin therapy compared with scutellarin or low level ultrasound alone treatment groups (Fig. 5, p<0.05). However, the increase is much less than sonodynamic therapy case, for which the ROS generation will be greatly enhanced when a sonosensitizer is used with ultrasound. Therefore the combined treatment produced enhancement is not through sonodynamic effect. In other words, scutellarin is not a sonosensitizer.

### Antitumor Effect *in vivo*

**Figure 6:**
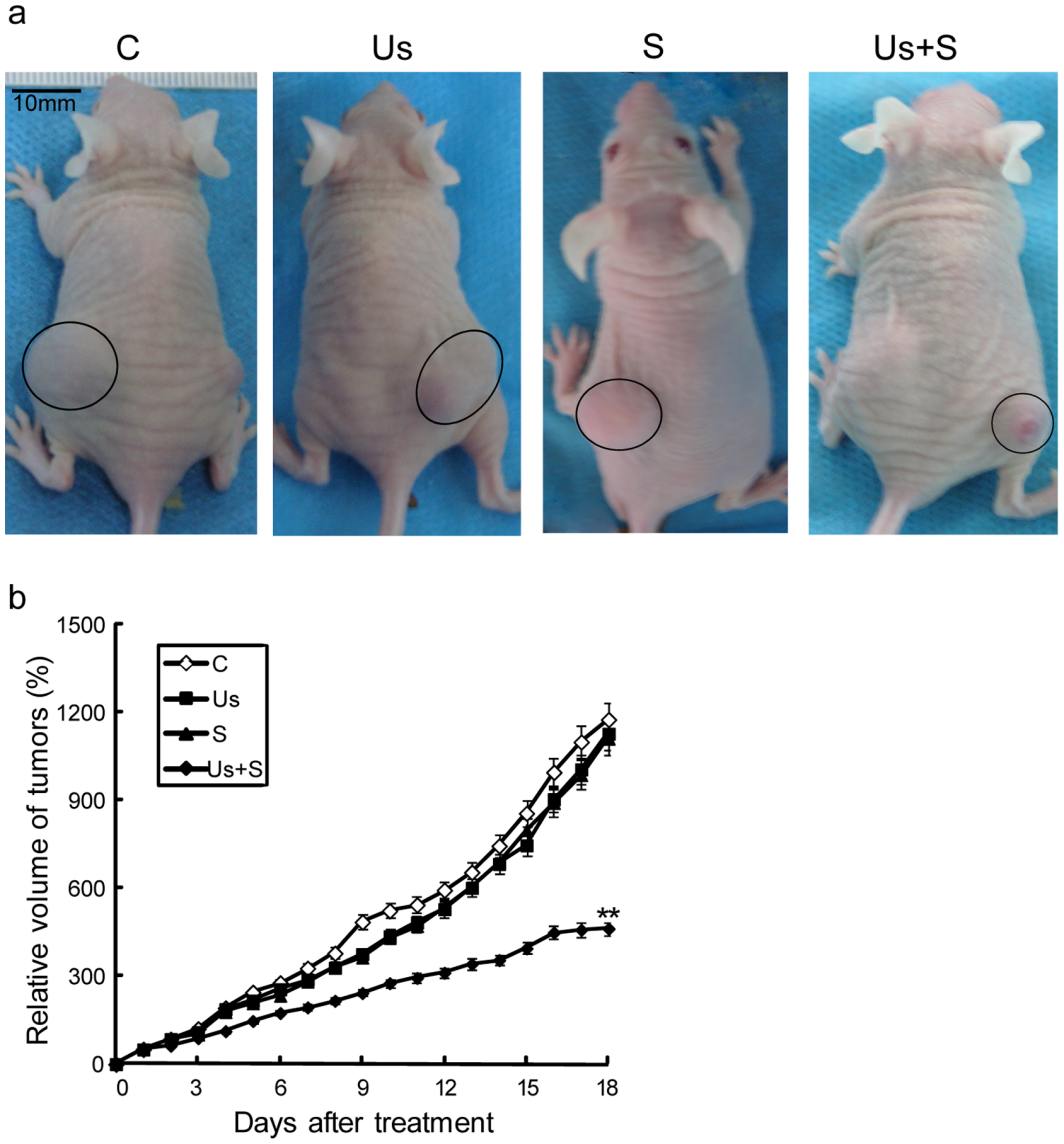
Inhibitory effect of *scutellarin* and ultrasound on tumor growth. (a) Gross view of a representative globular SAS xenograft tumor on the rear area of a nude mouse. The tumor mass in the C, S, and Us groups was more evident than that in the Us+S group. (b) Growth curve of SAS tumors. Relative volume (V_R_) of tumor growth was calculated as a measure of tumor growth for the C, Us, S, and Us+S groups. Data are mean ± SD values; n = 10 to 12 mice per group. **p<0.01 *vs.* control. Scale bar = 10 mm.

Based on the *in vitro* experiments the antitumor efficacy of *scutellarin* treatment against human tongue carcinoma cells can be greatly enhanced when it is combined with ultrasound, we speculated that the combined treatment also can produce increased anti-tumor effects *in vivo*. Indeed, from the in vivo treatment on xenograft nude mice model, the tumor volume in the Us+S group is much smaller compared to the C or S groups (Fig. 6a). Figure 6b shows the change in relative tumor volume with the number of days after each treatment. The results indicated that the S and the Us groups experienced no significant difference in tumor growth compared with the control group. In contrast, the Us+S group exhibited significant differences in tumor growth compared with the other groups at 18 d after treatment (p<0.01). The increase in relative tumor volume was greater than 1000-fold in the C, Us, and S groups, but less than 500-fold in the Us+S group. No significant differences were found among any of the groups in terms of mouse body weight.

### Transmission Electron Microscopy

**Figure 7:**
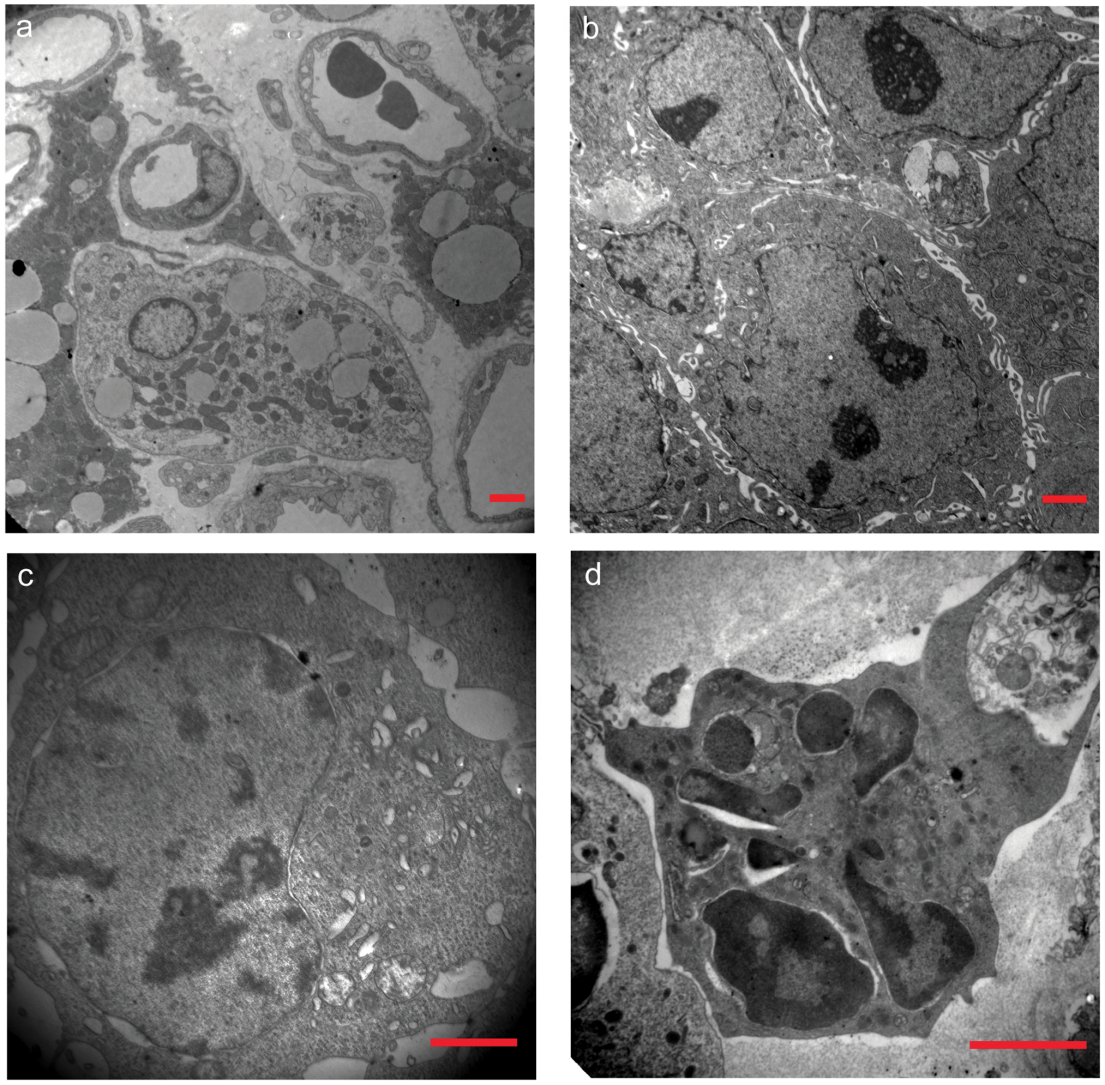
Transmission microscopy of ultrastructural changes in SAS cell xenograft tissue. Figures a–d show the C, Us, S, and Us+S groups, respectively. Scale bar = 2 µm.

TEM revealed that cells in the C group had many vascular structures, intact cell membranes, abundant cytoplasm, many normal mitochondria with well-developed cristae, and densely arranged chromatin (Fig. 7a). The cells in the Us group maintained an intact cell membrane but exhibited slightly swollen mitochondria, chromatin condensation, and occasional apoptosis, with apoptosis more apparent in the collagen-rich, reduced connections between the cells (Fig. 7b). Although cells in the S group did not show significant changes in size, some cells had begun the apoptosis process and had experienced mitochondrial swelling; nucleus cracking, albeit with no apparent nuclear condensation; and an increase in collagen content. However, the desmosome junctions between the cells could still be observed (Fig. 7c). In the Us+S group, the cell membranes remained intact but the cell chromatin had undergone significant changes; the cytoplasmic organelles had become sparse; several vacuoles, many small vesicles, and nuclear fragmentation with condensed nuclear chromatin had become apparent; and apoptosis activity had significantly increased (Fig. 7d).

### Cell Proliferation and Apoptosis *in vivo*

**Figure 8:**
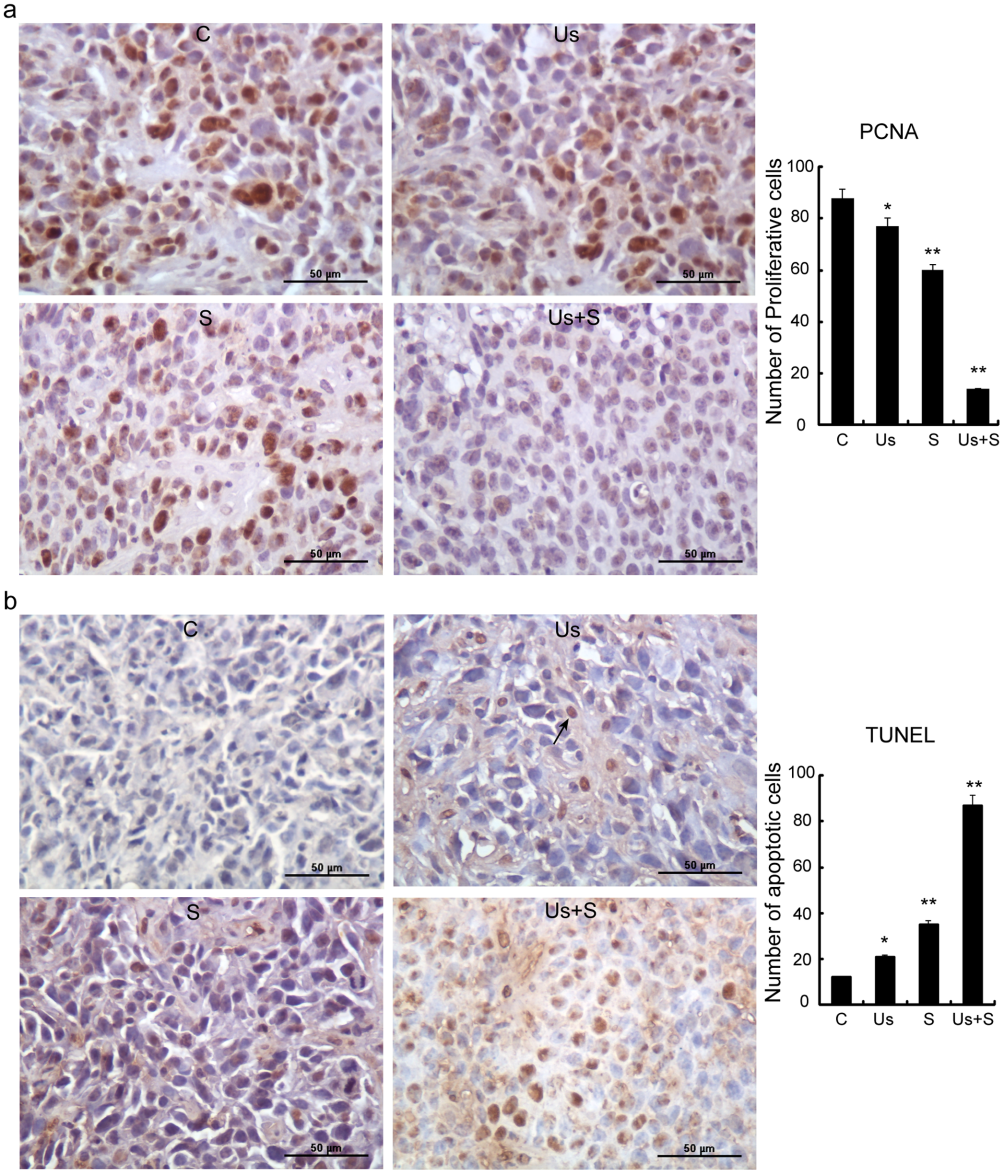
PCNA (a) expression and TUNEL staining (b) in tumors of C, Us, S, and Us+S groups. Numbers are mean ± SD values. Statistical significance was determined by one-way ANOVA Dunnett’s t-test. *p<0.05 and **p<0.01 *vs.* control group. Scale bar = 50 µm.

No significant differences in proliferation index were found between the C and Us groups. Compared with the C group, both the PCNA staining intensity and the cell proliferation index had significantly decreased in the S (p<0.05) and Us+S (p<0.01) groups (Fig. 8a). Little cell staining was observed in the Us+S group, with only remnants of small nests of tumor cells showing staining. As shown in Figure 8b, TUNEL assay to detect *a*poptosis *in vivo* revealed no stained cells in the C group. Cells in the Us group experienced some degree of apoptosis, with apoptotic cells visibly scattered in the transplant tumor, while apoptosis was significantly increased in the S group compared with the C group. Transplant tumor tissue within a large area of tumor cell apoptosis and necrosis could be observed in the Us+S group. The TUNEL score of the Us+S group was found to be significantly higher than that of the other 3 groups (p<0.01).

### MVD and LVD of Xenografts

**Figure 9:**
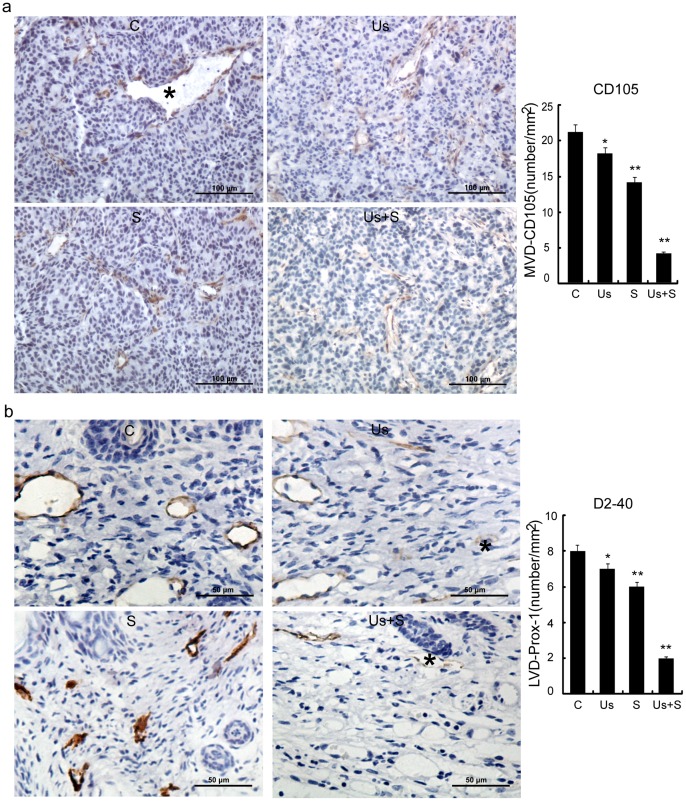
CD105 (*) expression (a) and D2–40 (*) expression (b) and microvessel density (MVD-CD105) and lymphatic vessel density (LVD-D2–40) for C, Us, S, and Us+S groups. Data are mean ± SD values. Statistical significance was determined by one-way ANOVA Dunnett’s t-test. *p<0.05 and **p<0.01 *vs.* control group.

The CD105 staining results revealed that the mean MVD of xenografts in the Us and S groups was lower than that of the C group and that the reduction in MVD was significantly greater in the xenografts of the Us+S group compared with that in the other 3 groups (p<0.01; Fig. 9 a). The results of D2–40 staining showed a reduction in LVD in the Us+S group compared with that in the other 3 groups (p<0.01; Fig. 9 b).

### MMP Level

**Figure 10:**
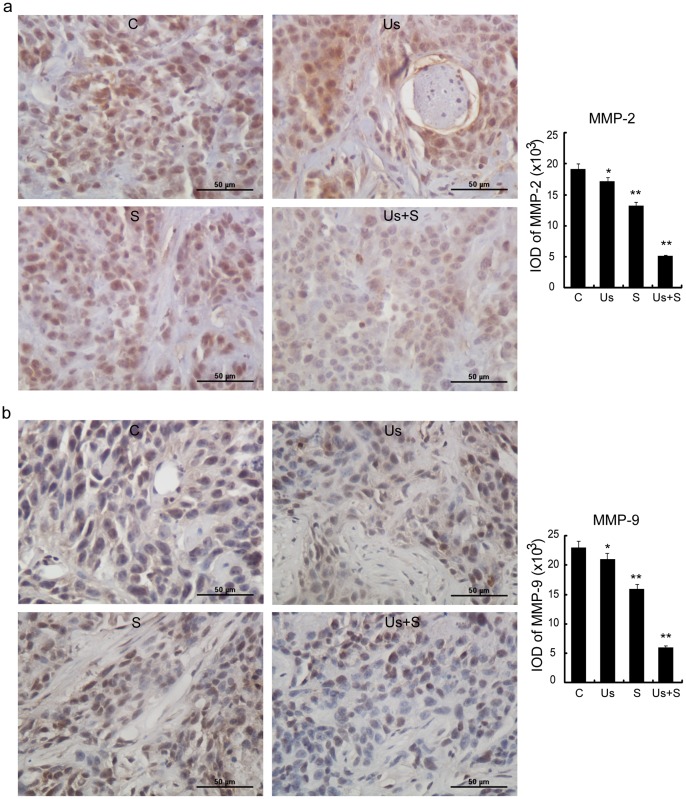
MMP-2 (a) and MMP-9 (b) expression of C, Us, S, and Us+S groups. Arrow: small cells. Data are presented as mean ± SD values. Statistical significance was determined by one-way ANOVA Dunnett’s t-test. *p<0.05 and **p<0.01 *vs.* control group. Scale bar = 50 µm.

As shown in Fig. 10, the mean expression levels of MMP-2 and MMP-9 in the cells of the Us+S group were significantly decreased compared with those of the other 3 groups. MMP-2 expression was observed in both large and small tumor cells (Fig. 10 a), while MMP-9 expression was observed primarily in large tumor cells (Fig. 10 b).

## Discussion

Ultrasound can induce structural and functional changes in tissues and cells. Among its many applications, it can be used to kill targeted cells or modulate cellular physiological/pathophysiological functions, whether used as a sole form of treatment or used concurrently with other treatments to achieve therapeutic goals [27]. Many studies have confirmed the ability of ultrasound to enhance the effectiveness of chemotherapy drugs [10], [13], [14], [28]–[31].

This study revealed that the combination treatment using *scutellarin* and low-intensity ultrasound has significant anti-tumor effect on human tongue cancer both *in vitro* and *in vivo*. Specifically, combined Us+S treatment was found to inhibit cell invasion, migration, and apoptosis; alter cell morphology; and decrease the number of migratory and invasive cells to a much greater extent than control treatment, indicating that ultrasound can enhance the efficacy of *scutellarin*-induced cell damage, cell growth, and cell motility. While low-level ultrasound or low-concentration *scutellarin* exhibits no significant antitumor effects when used alone, their combination treatment yields a cytotoxic effect sufficient to produce a significant therapeutic benefit.

At 18 days after treatment, tumor growth was observed to have been delayed by more than 50% in the Us+S group compared with the S group. Electron microscopy revealed that fewer changes had occurred in the organelles in the cells in the cells of the S group, while several changes in the cytoplasm had occurred later than they had in the nucleus. In the cells of the Us+S group, the number of desmosome junctions had increased, while the tumor cells showed typical apoptotic characteristics although the cell membranes had remained intact. Electron microscopy and optical microscopy revealed that the combined treatment had significantly inhibited angiogenesis and inhibited differentiation, proliferation, and transfer of tumor cells. In addition, MMP-2 and MMP-9 expression levels were found to be significantly lower in the cells treated with combination therapy, indicating that the treatment was controlling cancer cell invasion and metastasis [3].

Tongue cancer primarily develops through lymphatic metastasis. The study results indicated that combined treatment inhibits lymphangiogenesis in treated tumors, destroys tumor cells, induces apoptosis, inhibits MMP secretion, and inhibits tumor microvasculature formation, while low-level ultrasound or low-concentration *scutellarin* treatment alone yields little effect.

Ultrasound is known to promote membrane permeability, thus increasing intracellular drug accumulation. Previous studies suggested that the mechanisms by which ultrasound enhances the cytotoxicity of chemotherapy drugs is by increasing ROS generation [31], intracellular drug accumulation [32], and cell membrane permeability [10]. When the intracellular ROS level was measured to identify which mechanism is responsible for the findings, the combined therapy only produced moderate increase of the ROS level compared to *scutellarin*-alone or ultrasound-alone treatment, both of which generated ROS. The ROS generated from the combined treatment is much less compared to sonodynamic therapy, in which ROS generation is greatly enhanced. This finding indicates that the mechanism behind the enhanced anticancer effect provided by low-level ultrasound is not through the enhancement of ROS generation or a sonodynamic effect and *scutellarin* is not a nososensitizer.

Several researchers have hypothesized that ultrasound increases the intracellular drug level, leading to increased cell sensitivity to chemotherapy. Among them, George et al. reported that 1 h of tone-burst ultrasound increased cellular adriamycin accumulation [32], while Tinghe et al. reported that low-level ultrasound both enhanced the cytotoxicity of adriamycin to human ovarian carcinoma cells and promoted intracellular drug accumulation [13]. The results of Sundaram et al. indicated that the key mechanism of ultrasound-enhanced chemotherapy may be cavitation-generated free-radical production, which, by damaging cell membranes and promoting membrane permeability, increases intracellular drug accumulation [13], [33]. In contrast, Wu et al.’s research revealed that the shear stress in cells generated by ultrasound produces cell sonoporation, which temporarily “opens” cell membranes, allowing drugs to be delivered into cells [12], [34]. On the other hand, several experiments indicated that ultrasound does not increase intracellular drug accumulation but rather enhances therapeutic effects [13]; that is, sonication lowers the threshold of membrane rupture [14].

In our experiments, no evidence of sonoporation was found 1 h after the combination treatment, which suggests that the ultrasonic energy used was insufficient to cause irreversible sonoporation. We therefore hypothesize that the main mechanisms underlying ultrasound-enhanced chemotherapy are mechanical effects. Specifically, sonication increases the frequency and area of exposure of drug–cell contact such that the mechanical pressure generated at the contact site increases the probability of drug uptake into the cells, thereby enhancing drug absorption to produce better therapeutic effects.

Although some researchers argue that pulsed ultrasound may promote the spread of tumor cells and accelerate metastasis [35], our results did not show an increase in the metastatic ability of tumor cells, but rather a reduction in the extent of cancer metastasis. Likewise, no significant increase in the number of lung metastases was observed in a preclinical study of ablative exposure in a highly metastatic prostate cancer line implanted into the hind leg of mice [36], nor in the number of lung metastases in a study of high-amplitude ultrasound exposure on subcutaneous tumors implanted into the hind leg of mice [37]. Those findings lead us to speculate that the emergence of metastasis may be related to ultrasound intensity. Ultrasound-induced metastasis occurs only when the intensity increases beyond the threshold of cavitation. Both ultrasound (intensity, frequency, waveform, exposure duration, focal depth in tissue, and exposure methodology) and tissue (type and physiological status) characteristics affect treatment outcomes. Exposure to an identical form of ultrasound irradiation may produce drastically different effects on different tissues. Therefore, exploration of the mechanism of ultrasound-enhanced chemotherapy must be conducted according to experimental conditions, and ultrasound parameters must be screened for each case in order to achieve the desired therapeutic effect.

Low-frequency ultrasound can deeply penetrate targeted tissues and deliver energy to nonsuperficial objects. Ultrasound-activated pre-loading of drugs or reagents in specific tissues can lead to accumulation of high drug levels in target cells, which can reduce the dosage and concentration of reagents necessary to maintain or improve treatment efficacy. Both the *in vivo* and *in vitro* results of our study suggest that low-intensity ultrasound significantly increases *scutellarin*-induced inhibition of SAS cell growth, invasion, migration, and tumor vasculature formation. We also show that the combined low intensity ultrasound-scutellarin therapy on non-cancerous cell line (L6 rat myoblasts) has no significant cytotoxicity.

In general, low intensity ultrasound is a relatively safe, easy accessible, inexpensive, non-invasive, and non-toxic form of treatment that can also be focused to a specific region deep in the tissue. Therefore, it has great promise as a means of enhancing targeted drug delivery and site-specific treatment. More importantly, it can greatly reduce the dosage of chemotherapeutic agents necessary to yield a therapeutic effect, allowing for the reduction or even elimination of side effects.
